# The Deceptive COVID-19: Lessons from Common Molecular Diagnostics and a Novel Plan for the Prevention of the Next Pandemic

**DOI:** 10.3390/diseases11010020

**Published:** 2023-01-28

**Authors:** Dimitra S. Mouliou

**Affiliations:** 38500 Volos, Magnesia, Greece; demymoole@gmail.com

**Keywords:** COVID-19, SARS-CoV-2, emerging infectious diseases, molecular diagnostics, PCR, antigen test, antibody test, public health, preventive medicine

## Abstract

The COVID-19 pandemic took place during the years 2020–2022 and the virus, named SARS-CoV-2, seems likely to have resulted in an endemic disease. Nevertheless, widespread COVID-19 has given rise to several major molecular diagnostics’ facts and concerns that have emerged during the overall management of this disease and the subsequent pandemic. These concerns and lessons are undeniably critical for the prevention and control of future infectious agents. Furthermore, most populaces were introduced to several new public health maintenance strategies, and again, some critical events arose. The purpose of this perspective is to thoroughly analyze all these issues and the concerns, such as the molecular diagnostics’ terminologies, their role, as well as the quantity and quality issues with a molecular diagnostics’ test result. Furthermore, it is speculated that society will be more vulnerable in the future and prone to emerging infectious diseases; thus, a novel preventive medicine’s plan for the prevention and control of future (re)emerging infectious diseases is presented, so as to aid the early prevention of future epidemics and pandemics.

## 1. Introduction

In December 2019, a novel severe acute respiratory syndrome coronavirus 2 (SARS-CoV-2) emerged in Wuhan, China, leading to the disease named coronavirus disease 2019 (COVID-19), which was eventually portrayed as a pandemic [1]. Nowadays, it seems that COVID-19 has possibly become an endemic disease [2]. 

To date, scientific communities, international alliances, and governments worldwide have exerted considerable attempts to prevent, surveil, and manage SARS-CoV-2 dynamics and confirmed COVID-19 cases [1]. Public health measures were mostly based on quarantine and other general recommendations such as hand hygiene, mask use, and physical social distancing [3,4,5,6,7,8,9]. Other stricter community measures were the so-called «lockdowns» that limited activities or access to resources, facilities, or institutions, leading to generalized home isolation [10,11,12]. Moreover, a plethora of diagnostic tests and community mass-testing strategies were evident, in parallel with various vaccination platforms initially authorized for emergency use, and the so-called «COVID-19 vaccination certificate/pass», SARS-CoV-2 infection certificate/pass, or the SARS-CoV-2 negative molecular diagnostic test certificate/pass in some countries [2,13,14,15,16,17,18,19,20,21,22,23].

The COVID-19 pandemic shaped the era of molecular diagnostics, because, in reality, it was the first attempt at applying molecular diagnostics worldwide on a large scale [21]. Nevertheless, this pandemic has undeniably provided the scientific communities and populaces with multifarious and unique experiences. At this stage, where the initial series of COVID-19 pandemic waves have become a past reality, it is time for scientific communities to reassess the overall pandemic and identify the lessons it has taught us, especially in the molecular diagnostics field. Doubtlessly, we are now at the post-pandemic stage; thus, we should ascertain the current existing societal weights and measures so we are capable of preventing and managing the next emerging infectious disease before it becomes the next pandemic. 

The first part of this critical perspective highlights the most crucial issues that emerged during the COVID-19 pandemic and how they are related to molecular diagnostics, with a critical evaluation, instructive aspects, and potential solutions. Furthermore, the near-future public health reality is estimated and contemplated, based on pre-pandemic and post-pandemic knowns, and finally, a novel preventive medicine-based plan is illustrated and discussed for the prevention and management of future emerging infectious disease epidemics and pandemics.

## 2. Current COVID-19 Pandemic-Derived Molecular Diagnostics’ Facts, Concerns, and Critical Evaluation 

### 2.1. Molecular Diagnostic Testing and Terminology

Historically, this was the first time that most populaces worldwide came so close to molecular diagnostics at such a great extent; the COVID-19 pandemic was the hallmark for self-diagnosis via diagnostic tests by non-experts. As a result, several issues should be taken into consideration and reassessed appropriately. First and foremost, it must be highlighted, that, according to the molecular biology principles, «molecular tests», include all nucleic acid amplification tests (NAATs) such as the widely performed polymerase chain reaction (PCR) assay for the genetic identification of SARS-CoV-2 and all serological tests, either for antigen or antibody detection, such as the lateral flow immunoassays (LFIAs) that society regarded as ‘rapid tests’, as well as the laboratory antigen and antibody detection tests, such as the widely preferred enzyme-linked immunosorbent assay (ELISA) [21,22,23,24,25]. All these testing platforms and several others have already been applied for managing the molecular diagnosis of infectious diseases, while common and new ones were applied for the identification of SARS-CoV-2 [26]. Nevertheless, it is evident that various sources revealed an incorrect discrimination of PCR as being the sole molecular test compared to LFIAs [27,28]. Several studies in the literature make such a discrimination and, as a result, the media and society followed this inaccurate information. 

Apart from basic conventional laboratory diagnostic methods, the COVID-19 pandemic mainly presented the opportunity for more rapid nucleic acid detection tests to be designed, and those based on LFIAs allowed for a better, precise point-of-care and immediate identification of a pathogen [29]. As a result, there is no realistic logical argument for such a discrimination to be made. In the near future, loop-mediated isothermal amplification (LAMP) and recombinase polymerase amplification (RPA) may be widely performed for the rapid point-of-care identification of several pathogens [30]. Other rarely commercialized but newfangled technologies include clustered regularly interspaced short palindromic repeats (CRISPR), mass spectroscopy (MS), electro-chemical immunosensor and nanosensor platforms, rapid point-of-care microfluidic-based assays, surface-enhanced Raman spectroscopy (SERS), and other machine learning approaches [31,32,33,34,35]. It is estimated that such novel or other new molecular diagnostic methods will be widely disseminated in the near future for the detection of future (re)emerging infectious agents; thus, the terminology of molecular diagnostics must be urgently and appropriately corrected. Additionally, there is evidence that ‘molecular’ testing is compared with ‘rapid’ testing [27]. Compared to a classical laboratory method that requires hours for a result to be taken, a ‘rapid’ test characterizes the speed in which the performer is able to receive results. On the contrary, the term ‘molecular’ describes the test target or type. In reality, both these tests (rapid and molecular) are included in molecular diagnostics, so again this comparison has no logical scientific argument [26]. Moreover, the term ‘rapid’ is not a scientific method to be reported in the final test result, but, unfortunately, various scientific and media data reveal that both scientists and societies are misinformed, ignoring that the precise method is LFIA.

Finally, another piece of misinformation was the discrimination of self-tests and rapid tests in some countries, even if both of these two tests were actually based on LFIAs [36]. Again, it is evident that both of these two tests are the same. The term ‘self’ cannot be compared with the term ‘rapid’ since these two words describe something different. For example, if a laboratory scientist performs a PCR assay to his sample, this will be a ‘self-test’, regardless of the complexity, target, or speed of the assay. 

All these basic molecular diagnostics’ terminologies are fairly simple, so they can be misstated across populaces and nations. The COVID-19 pandemic has undeniably introduced to society the era of molecular diagnostics and new, novel, favorable molecular testing strategies will be seen and prevail in the near future. Therefore, such misinformation regarding terminologies should not have existed not only in the scientific literature data but also in the media and anywhere else. 

### 2.2. Molecular Diagnostic Testing and Its Role

Doubtlessly, the role of molecular diagnostic tests should be reevaluated in the context of case definition. The initial role of the PCR procedure was to make thousands of millions of (amplify) copies of a particular section of nucleic acid, even in extremely small accounts [37]. Nowadays, this assay has become the centerpiece of the molecular detection of various infectious diseases, even for COVID-19 in the version of reverse transcription PCR (RT-PCR) as SARS-CoV-2 is an RNA virus [2,22,38,39,40,41,42]. Nevertheless, and via its mechanism, it is revealed that a PCR assay detects a sequence of nucleic acid, and, as a result, it cannot distinguish between active genes and genetic fragments [2,22]. 

LFIAs and classical laboratory tests work with a similar tactic when they identify their targets (referring to the antigens and antibodies), but there is no multiplying procedure in these methods. However, it seems unclear if serological methods could detect antigenic/immunoglobulinic fragments, so as to lead to a final false-positive test result [2,22]. 

In other words, it is not possible for common molecular diagnostic tests to differentiate between active and inactive pathogens since their initial sole role is to identify the presence of a biomolecule. Compared to the initial mechanism of action and role of the PCR assay, the World Health Organization (WHO) defined a confirmed case of COVID-19 to be anyone with a positive NAAT (mainly referred to RT-PCR as this assay was widely performed during the pandemic) regardless of clinical or epidemiological criteria [43]. As a result, a critical perspective doubted the real existence of COVID-19 asymptomatic patients since, by being a patient, it is meant that the person is ill; thus, the signs and symptoms of a disease are evident [43]. 

Nevertheless, the scientific community has already known, from the pre-pandemic years, that a PCR cannot be compared with the role of a clinical physician that identifies a disease, but instead, it recognizes the genetic fragments. Therefore, this initial recommendation regarding COVID-19 testing should not be evident and it should be reevaluated and restated at a societal level not only for COVID-19, but also for future infectious diseases. Additionally, by screening asymptomatic patients without the history of a COVID-19 confirmed case contact, there is a high possibility of a test being inaccurate, giving rise to several consequences for the tested individual and the accuracy and acceptability of this testing assay as well [2,5,43]. 

However, even in Ag-RDTs, there should be a strict adherence to the instructions of a test kit so professionals can avoid false test results that can occur for multifarious reasons, otherwise from the overuse of public health resources (such as in mass testing). The dissemination of misinformation in regard to these tests could have resulted in multiple scientific biases; also, it could lead to having a tremendous negative impact on society [2,22,44]. 

Moreover, concerning the identification of immunoglobulin during the infection by a pathogen, it should doubtlessly be highlighted that by detecting antibodies (that are the host’s response to a pathogen), the real pathogen is not detected. Moreover, false test results seem to be more common in serologic assays for multifarious reasons [2,22,43]. 

In conclusion, it seems unscientific and illogical for confirmed infected cases to be globally reported via nucleic acid or/and antigen tests in different nations because, by that way, there is no common denominator regarding the testing target. Even the various PCR test kits’ protocols are based on different Ct values; thus, a case that has been found positive in one test can be found negative in another test kit. Undeniably, such issues trammel the scientific community from making precise hypotheses for the prevention and control of emerging infectious diseases. 

### 2.3. Molecular Diagnostic Testing and Quantity 

Another fact regarding the application of molecular tests in the diagnosis of emerging infectious diseases’ transmission is the quantity issue. There have been conducted and published several research articles about the so-called number of cycles (Ct), its appropriate threshold value, and the correlates of viral infectivity for SARS-CoV-2 during the pandemic [45,46,47,48,49]. Yet, others have related the Ct value with viral infectivity, whereas others have revealed the opposite; thus, the Ct issue is a controversial topic. 

Furthermore, there exist studies which attempted to compare Ct values in symptomatic and asymptomatic cases [50,51]. It has also been discussed that the usefulness of the PCR assay in COVID-19 patients can be diminished by simplifying its test result as positive or negative and that the Ct value (when the test result is positive), can assist in decision-making when interpreted in the clinical context of patients [52]. 

Nevertheless, one should bear in mind that a test is performed in a random sample received at a random point of time from a random case. Moreover, sensitivity and specificity are inversely related; when sensitivity elevates, specificity seems to be lower, and vice versa.

Additionally, SARS-CoV-2 tissue-specific infectivity dynamics are different for various mutants, as the initial virus would mostly be present in low respiratory tract samples, whereas the current mutants are highly infective for the upper respiratory tract [2,43]. Taking into consideration the existence of false-positive and false-negative test results, the fact that SARS-CoV-2 can be reverse-transcribed and integrated into the human genome in cultured human cells, and also that it can be expressed in patient-derived tissues, one should argue that infectivity cannot be detected via Ct or the overall test result [2,5,53]. 

Since viral residues can persist for a long time after an initial infection, the necropsies in post-COVID-19 decayed cases could have a molecular test result positivity, but this result could not precisely depict the reality. Because deaths due to COVID-19 were counted to be all the cases with an initial positive test result and a following death (related or unrelated to COVID-19), the real COVID-19 fatality rates are now estimated. Yet, it will be extremely difficult for the accurate mortality rates to be depicted via a sole molecular test because the golden standard PCR cannot differentiate between active and inactive viral segments.

Furthermore, it is evident that most people have been vaccinated against COVID-19 and, as seen in other pre-pandemic pathogens, vaccinations can result in false test results [22]. Therefore, the Ct issue should not exist. Even if genetic segments can persist for long time post-COVID-19, the quantity (referring to the result of a test) can nowhere be applicable. That is because the virus is transmitted but its genetic fragments cannot be transmitted, at least according to the existent literature data. Moreover, it is worth mentioning that the Ct value is highly affected by various inhibitors, thus a high Ct that has actually arisen by potential inhibitors does not depict a low viral load [2,22]. Sadly, laboratory errors regarding the viral quantity affect public health and epidemiology as well. 

It should not be ameliorated that several PCR assay test kits’ protocols are based on various Ct values and there does not exist any common denominators [2]. This fact by itself highlights that the specific Ct value that a test was positive for does not really matter and that it should not be of such scientific concern. Additionally, people should not be informed about this useless information regarding their personal Ct test result as they attempt to make personal comparisons after infection. Therefore, society is again misinformed. Doubtlessly, the Ct role is uniquely a scientific issue far away from the direct positive or negative test result. It is solely required for diagnostic purposes on the application of molecular diagnostic testing for the identification of a patient at the molecular level.

Regarding the Ag-RDTs, a similar aspect should be taken into account concerning the quantity of the detected antigens; it should be highlighted that the overapplication in mass testing can have a negative impact on society. Undeniably, similar aspects should be followed by future novel molecular diagnostic testing procedures as well.

Finally, and far away from the quantity at a nanoworld’s level, the realistic test quantity is of vital importance for cases requiring urgent management and treatment [1,2,22]. Retesting via performing a different test kit, resampling and sampling a different tissue (e.g., nasopharyngeal vs. bronchoalveolar lavage sample), and retesting but with a different testing target (e.g., antigen vs. nucleic acid vs. antibody) obtains a combination of test results in an ambiguous case of a hypothesized false test result; these are some major tactics which ought to be globally highlighted for front-line physicians [1,2,22]. 

### 2.4. Molecular Diagnostic Testing and Quality

The quality of a molecular test is depicted by its specificity and its sensitivity. During the pandemic, several scientific data highlighted that there are several reasons that can lead to false-positive and false-negative COVID-19 test results [2,22]. Taking into account that tests have been performed in cases with COVID-like signs and symptoms, it is again difficult to estimate the real COVID-19 mortality rates [22,54]. For example, factors including nonclear places, sampling/handling contaminations, temperature, deficient sampling, suboptimal processing/RNA extraction, technical reasons (e.g., prime-dimers, short/nonspecific primers, probes, and fluorescence), Ct cutoff value/control in different test interim guidance, cross-contaminations in sampling, handling, laboratory (especially in 2-step RT-PCR), inhibitors (mainly bloody and viscous samples), cross-reactions with other pathogens/tissue nucleic acids or SARS-CoV detection, SARS-CoV-2 nucleic acid degradation, inactive/residual SARS-CoV-2 genetic sequence detection, and the ongoing viral mutants can all lead to false RT-PCR assay test results [2,22]. Moreover, generally regarding antigen and antibody tests, the non-clear place, sampling/handling contaminations or poor sampling/ sampling degradation, time of sampling, humidity and position for LFIAs, sample viscosity, temperature, time to evaluation (early or late reading of the test result especially for LFIAs), destroyed cassette, viral mutations, cross-reactions with other antigens or cross-reactions with other antibodies, the so-called Hook effect for LFIAs (high antigenic load and subsequent false-positivity in LFIas), antibody production affecting factors (e.g., age, sex, diet, smoking, adjuvants, vaccines, genetics, etc.), SARS-CoV-related antigen/antibody detection, vaccination against COVID-19, SARS-CoV-2 inadequacy, exogenous/endogenous other antibodies, IgG positivity long after initial infection or positivity due to viral shedding or viral residual genetic sequences, generally the late or early test implementation regarding the infection window period, exogenous factors for LFIAs (e.g., high concentrations of nasal spray, chemical substances, or ions), and endogenous factors (e.g., Ig-drugs or blood-impurity-derived substances for LFIAs) can all affect the final test result and lead to inaccuracies and false antigen/antibody test results [2,22]. Additionally, it can be concluded by certain nucleic acid test kits, such as the PCR, that some tests are highly affected by the tissue type from where the sample is received, and especially if underlying disorders and diseases exist [2,22]. Moreover, it has already been revealed that serologic assays can be affected by underlying chronic inflammatory conditions (referring to autoimmune and non-autoimmune chronic diseases), preexistent infection by Epstein–Barr virus (EBV), cytomegalovirus (CMV), dengue virus (DENV), human immunodeficiency virus (HIV), or/and a history of other conditions such as hepatitis, malaria, syphilis, angiitis, hypergammaglobulinemia, HLA-DR antibodies, human anti-animal antibodies (HAAAs), immunoglobulin-based immunotherapies, as well as the biotin supplements regarding the ELISA serologic tests [2,22]. All these factors were evident before COVID-19 resulted into a pandemic. 

On the contrary, there have existed several case reports that illustrate false-positive and false-negative test results but they do not provide important information, such as the brand name of the test kit that the false result had occurred or the overall preexisting medical condition of the reported case. One can find several case reports in the current literature, with such content describing false tests either regarding nucleic acid detection or antigen and antibody detection [55,56,57,58,59,60]. As a result, one cannot search for the specific performed test kit protocol to read the limitations of the performed test so as to identify the precise molecular reason of the false test result. Additionally, and hypothesizing that the test kit protocol is reported, if there is not enough information for the medical history of a case with a false test result, then, again, the definite reason of inaccuracy cannot be identified. The false test result is mainly a molecular issue; to my knowledge, there is no such case report with its authors to discuss the reasons of the reported false test result through the performed test kit protocol and the preexisting medical conditions of the case at a molecular level because the false test result case reports are mostly discussed at a clinical level. Therefore, one can see that, during the pandemic, several case reports have, basically, been mistakenly reported. It is extremely important for test kit protocols to be thoroughly comprehended before reporting a case with a false test result. One could guess that in the vast majority of the reported cases with false test results, it was not the hypothesized error in the specific test kit that led to false results but the wrong implementation of that specific test. Doubtlessly, there exist various reasons thoroughly described in each test kit protocol that highlight a potential testing incapability. As a result, it is not the specific test which should be accused but the performer of the test that possibly could have not thoroughly read the test kit protocol. Scientists ought to have accurate and precise data so as to draw further conclusions for any event. These facts, concerning the case reports with false test results, must not be evident in future emerging infectious diseases. 

Another fact regarding false test results is that most studies are based on the incidence of false-positives and false-negatives by performing tests at a mass level, and no study is based on the etiology of the incidence of a false test result. Most studies generally analyze the time period from the initial viral infection to test positivity or/and the viral load frame. We know that nucleic acid tests detect genetic sequences from active/inactive viral genes, but Ag-RDTs are more sensitive in symptomatic compared to asymptomatic cases. Therefore, one could argue that in reality, the Ag-tests are more likely to reveal viral activity since positivity in a nucleic acid test in parallel with a negative Ag-test could mean that the nucleic acid is inactive and therefore unable to produce peptides [61]. Yet, regarding the false test results, it would be wise—at a biochemical level—to perform testing in targeted groups based on the limitations of each test kit’s protocol. For example, there could be implemented a test kit where the results among those with no underlying medical condition vs. those with chronic inflammatory conditions are studied so as to reveal the exact molecular reason of the incidence of false test results in those with medical issues, consequently highlighting the findings to the scientific community. Such issues should have taken place during the pandemic to obtain evidence and finally prevent false test results in future emerging infectious diseases, epidemics, and pandemics. Doubtlessly, molecular diagnostics and testing assays are our golden future; thus, it is required that precise and thorough studies be conducted so the scientific community can prevent and manage future infections, as well as for increasingly accurate novel diagnostic tests to be designed. 

**Figure 1:**
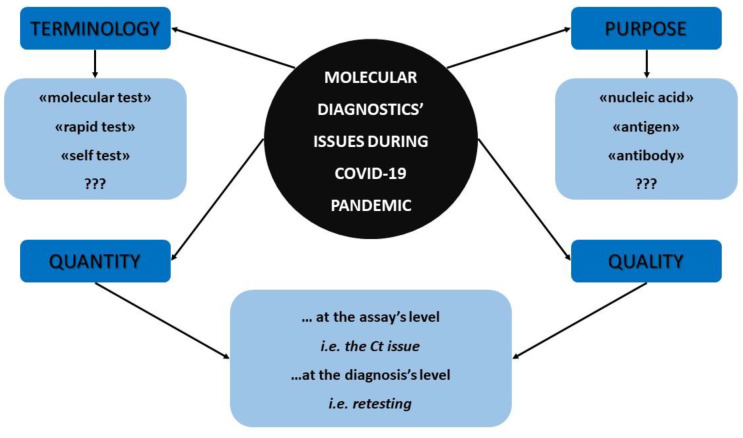
The major molecular diagnostics’ concerns that arose during COVID-19 pandemic.

Figure 1 summarizes all the aforementioned molecular diagnostics’ facts and concerns that were previously discussed which emerged during the COVID-19 pandemic and that should be reassessed by the scientific community. 

## 3. From Current to Future Coming Public Health and the Molecular Diagnostics: The Expert’s Estimation

The COVID-19 pandemic has widely introduced society to some major public health maintenance policies. These novel public health continuity strategies include rapid molecular self-diagnosis with point-of-care approaches, the entrance of the mRNA technology through vaccination against COVID-19, the imposition of the so-called lockdowns, and the certificate/pass in order for someone to obtain movement freedom [16]. The quarantine for the infected cases and their close contacts as well as mask-use were historically familiar to society from previous pandemics [3,4,5,6,7,8,9]. 

Society also became familiar with «post-disease syndrome», which was something novel for such flu-like emerging infectious diseases. In reality, we were supposed to know the post-infection post-asymptomatic period of the disease because of i.e., the so-called HIV, but it is supposed that HIV has to be active, replicative, and capable of transmission all the time after the initial infection [62]. The difference with SARS-CoV-2 is that the initial infection is not followed by the presence of an active virus as being a whole active pathogenic molecule, despite the ‘long-COVID’ phenotype. More studies are required on SARS-CoV-2 spike proteins’ serum titers post-infection. Even if SARS-CoV-2 can be reverse transcribed and integrated into the human genome in vitro, and even if the combination of the current data illustrates the possibility of the long presence of viral genetic sequences in certain cases with prolonged test positivity, there is no transmissible active virus as a whole molecule after the infection [22,53]. In parallel with the long-COVID syndrome, it has been revealed that some post-vaccination spontaneously emerging adverse conditions can be symptomatic for a long time, resembling a condition known as «post-SARS-CoV-2-vaccination syndrome» [63].

So, taking into consideration both long-COVID and the long-vaccination-against-COVID syndromes, one can see that, in the near future, populaces will be vulnerable but, in reality, the social vulnerability had been depicted during the pandemic period [64]. Additionally, it is evident that immunomodulatory approaches, especially monoclonal antibodies and related peptides, have come to the forefront as a major tactic in managing cancer, inflammatory conditions including autoimmune diseases, infections, and other health issues. Immunotherapeutics have been applied in COVID-19 as well [65,66]. It was evident from the pre-pandemic years that currently we have therapies and drugs but with no specific ‘disease’ label, as several therapeutic strategies and drugs can target and be applied to more than one disease; the pandemic also proved that fact. For example, a mono-clonal antibody studied in multiple sclerosis (MS) has been studied in COVID-19 as well [67]. Doubtlessly, current lifestyle affects genetics to a large extent, and as climate change is progressing, new emerging infectious diseases will arise or past infectious pathogens will appear again [2]. However, since immunomodulation may be our golden future in managing several medical conditions, that sole fact already takes for granted that a large percentage of the future population will be immunosuppressed, hence extremely vulnerable to infectious pathogens. Moreover, the widespread Western pattern diet (WPD) (or standard American diet (SAD)) that contains high amounts of processed foods, red meat, high-fat dairy products, high-sugar foods, and pre-packaged foods makes humans more acidic compared to the natural alkaline vegan and fruitarian diet. As a result, the internal body’s acidic conditions weaken the immune system and they make it more vulnerable to chronic inflammations; therefore, all parameters highlight that in the future, public health will be very vulnerable [68,69,70].

One could argue that maybe the lockdowns could prevent future pathogens’ widespread transmission and their extremely harmful effects on future vulnerable public health, but current society cannot shift from one time to another in such conditions. These events require such different societal bases. Maybe the global tourism pauses and the intercountry movements ceasing, along with the global general home isolation of all people and all previous actions for a period of time, i.e., a whole month, could draw to an end the COVID-19 pandemic. Maybe, by that way, the strict recurrent lockdowns and mutants’ pandemic waves would not have taken place and the vast majority of future emerging infectious diseases could fully disappear and not result in endemic diseases. Doubtlessly, most of the current face masks were not initially designed to prevent such infections, especially the viral ones, and they can have several side effects after prolonged use [6].

Nevertheless, undeniably, safe vaccines will be needed. May reverse vaccinology favorably contribute to inducing short-term immunity for future pathogens [71,72,73,74,75]. The short-lasting presence of antibodies may prevent the phenomenon of antibody-dependent enhancement (ADE) and even false test results. Multi-epitopic vaccines should be well-studied as it seems wise to induce immunity against several pathogens at one time; it is similar to the multiplexed molecular assays as they have more than one target at a time. In the near future, it is estimated from several aspects that the term ‘multi’ will be the golden ideal in medicine. However, people that receive new drugs and novel product injections should totally be monitored, and that is evident even from the vaccines against COVID-19. Prospective pharmacovigilance and the long-term monitoring of vaccinated recipients should be a public health priority for current and future vaccines, and strategies to monitor vaccinated people should have been evident from the very beginning of the vaccination campaigns in this COVID-19 pandemic. That is, the SARS-CoV-2 spike protein hypothesis highlights the need for vaccinated people to be safe and prevent any post-vaccination adverse events since the literature reveals that there exist major risk factors for such adverse occurrences [63,76]. For example, it was already known that multiple vaccine-induced immune-boosting strategies can weaken the immune system; it could even be hypothesized by that, nowadays, even vaccinated people with several vaccine doses can have severe COVID-19 and, as a result, even the less harmful mutants can be severe for some people [63]. Doubtlessly, herd immunity is not evident, even in more than a year post-vaccination year. Moreover, the random incidence of post-vaccination sudden adult death syndrome (SADS) among athletes has been reported and, doubtlessly, it requires a further investigation as the SADS was randomly reported in cases via myocarditis-induced events [63,77,78]. Both previous facts highlight that the scientific community should further investigate all the adverse events and provide explanatory hypotheses. Such studies highlight the need for defibrillators to be publicly available and ready to be used by anyone, as well as related educational courses must be freely available for all people. Additionally, cardiopulmonary and neurological and other critical examinations could be made freely available for people so as to prevent possible adverse events. Doubtlessly, public health should totally be monitored; specific point-of-care molecular diagnostic tests can be designed for these purposes. However, it has been discussed that inflammation already existed in society; thus, it is of vital and also of scientific importance for adverse events to be totally investigated in healthy people [63]. Undeniably, the promising and favorable RNA world has much more to offer in medicine.

All previously discussed facts will be evident in our near future society; hence, it is required that all these facts be taken into consideration in the case of the appearance of future emerging infectious diseases. SARS-CoV-2 has taught us that some pathogens have some similar antigenic sites and, in reality, there is only one thorough study that reveals such hypothesis and overall evidence for SARS-CoV-2 and other pathogens [79]. Yet, this fact could somewhat be hypothesized from the SARS-CoV-2 false test results that have occurred in relation to SARS-CoV-2 and other pathogens [2,22]. Nevertheless, it is of vital importance for such studies that reveal the common antigenic sites amongst pathogens to be evident for the future emerging infectious pathogenic agents from the very beginning of their occurrence. Why?

(1) First, there may exist a potential pre-existent immunity due to these antigenic sites of already known emerging infectious pathogens; thus, populaces that had faced these previous pathogens may be less vulnerable to the new emerging infectious disease. Therefore, the overall control and management of the new disease will be based on such evidence for the public health of the populaces worldwide. As a result, restrictions, vaccinations, immune pass/vaccination certificates, lockdowns, or other public health maintenance strategies will be so different amongst nations due to the variances in national vulnerability. Hence, we speak about a required «global immuno-blueprint» with data regarding previous exposure to emerging infectious pathogens (and their potential genetic residues in people as well) and the currently existent immunity. The global immunological blueprint can possibly prevent the ADE phenomenon and foresee the vulnerability of each populace to new (re)emerging pathogens. Of course, such evidence should be assessed in parallel with the societal physical condition, meaning the rates of underlying medical conditions in each populace. Nevertheless, the global immunological blueprint should be available for every immune-related aspect and not only for pathogens’ history amongst populaces. That way, the overall susceptibility of a populace to a potential severity of future (re)emerging infectious diseases will be highly prevented. This global immunoblueprint is generally essential for all aspects of preventive medicine and must be immediately evident and free for scientific community governances and other authorities worldwide.
(2) Second, it was previously stated that the scientific community has already been armored with effective therapeutics and that currently drugs can show favorable results for more than one disease. Therefore, known drugs that had previously targeted the anti-genic sites of known pathogens can immediately be in the front line for the physicians. An on-the-spot management of hazardous emerging infectious diseases can be achieved until the precise drugs are evident, or these drugs can even be administered as second-line drugs for those who are not able to receive the first-line strong drugs. New novel and more effective drugs are becoming rapidly evident based on the common anti-genic site and the already known molecular mechanism of action.
(3) Favorable vaccine platforms can be rapidly designed and safely administered since they will be based on these common antigenic sites between pathogens. That way, a vaccine can target more than one pathogen via a sole antigenic site. Of course, with one common antigenic site, one can target both pathogens, thus resulting in a lower activation of the immune system and not double (or more) as immune boosting. Additionally, lower serum overall antibody titers can be accomplished, thus the serum will be less sticky, and apart from the overall health benefits, false test results will be lessened. In this way, we speak about multitargeting epitope vaccines, compared to the now studied multi-epitope ones, that could potentially harm the initial power and dynamics of the human immune system. These vaccines can have the accurate most important epitope of a pathogen along with a shared one with another vaccine. As a result, the immune system can tackle both or more pathogens with a sole antibody type. However, SARS-CoV-2 has taught us that this vaccine-targeted antigenic site should be less capable of mutation; thus, the initial protein (that the pathogen uses to enter into a cell) may not be targeted because even the spike protein has accumulated several mutations compared to the other viral proteins.
(4) Last but not least, false test results that arise due to cross-reactions amongst pathogens will be prevented. If the scientific community reveals a pathogen’s shared anti-genic sites with the already known pathogens at a very early stage, more accurate, specific, and sensitive molecular tests will be designed that do not target such common antigenic sites. By that way, there will be no false tests because of cross-reactions amongst pathogens. If such pathogenic common antigenic site-based studies were largely evident for SARS-CoV-2, the condition of the pandemic condition would be much different.

**Figure 2:**
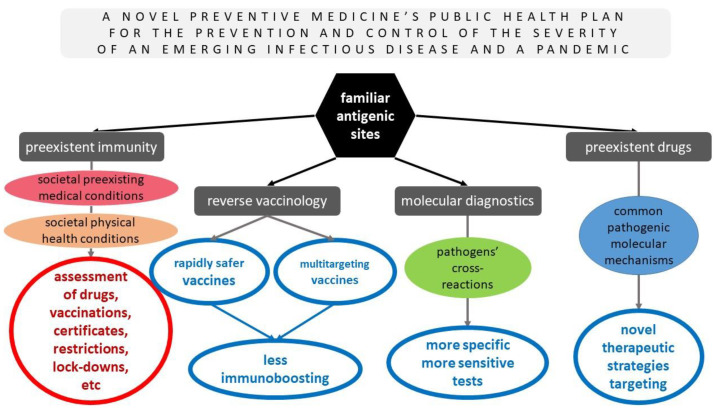
A novel preventive medicine’s public health plan for the prevention and management of the severity of future (re)emerging infectious diseases based on the hypothesis of the already known pathogens’ antigenic sites and their advantages.

Such sophisticated and wise strategies must immediately be performed on the basis of preventive medicine and public health for the prevention and control of the severity of future emerging infectious diseases. Figure 2 summarizes the pros of the novel preventive medicine’s public health plan for the prevention and management of the severity of future (re)emerging infectious pathogens that were previously discussed thoroughly and based on the hypothesis of the already known pathogenic sites that were evident in other pathogens but were revealed in SARS-CoV-2 as well, along with their advantages, for the potential common antigenic sites of future emerging infectious pathogens with some already studied ones.

Taking into account all the previously discussed issues, it is evident that the current post-pandemic new reality is a more vulnerable normality and more prone to new emerging pathogens. Even if the newly encountered pathogens are not as harmful, most of post-pandemic populaces already show vulnerability because of the previously discussed facts. The molecular diagnostics’ era has already shaped public health and will completely be in the frontline in the near future, especially at a self-diagnostic level. The rapid antigen self-diagnostic tests (RASDTs) that were initially disseminated to some nations, in the context of the identification of SARS-CoV-2, were our scientifically known Ag-RDTs. Unfortunately, research via a web-based questionnaire showed that 2/3 of the responders characterized self-tests as unreliable and 2/5 reported them as dangerous [36]. It is estimated that asymptomatic mass testing with its high rates of misdiagnosis—along with the overall fear of the pandemic—led to such societal aspects. Additionally, another study revealed that unvaccinated people were more likely not to receive the vaccine as a response to strict government policies [13]. Possibly the overall strict public health maintenance policies in some nations led several people to experience negative thoughts about self-testing and even be afraid of it. Nevertheless, it is of vital importance for people to be well-informed about the crucial role and the importance of self-testing, especially for emerging infectious pathogens. Self-diagnosis is the reality of the future of public health and it can prevent severe conditions, especially for vulnerable and immunosuppressed people. Moreover, molecular diagnostics should be applied in self-diagnosis via highly specific and more sensitive methods that will provide rapid but efficient point-of-care and low-cost diagnostic test results. Doubtlessly, they need to be alluring for non-scientists to buy and precisely perform them. It is worth mentioning that biotechnology has made extreme progress and there already existed some e-applications which are in parallel with the performance of RASDTs or other self-sampling but professionally diagnostic tests during the COVID-19 pandemic, so probable SARS-CoV-2 carriers can be immediately isolated and stay at home and not spread the infectious agent to the others; thus, public health could be very well-equipped in the near future. 

Several concerns regarding the Ct threshold value of the RT-PCR during the COVID-19 pandemic vastly highlighted the urgent need for novel future molecular diagnostic tests to be designed on the basis of a binary test result. Scientifically speaking, it is irrational for people to be compared on the basis of the random viral load of their random sample in such manners. The only thing that it is needed is a positive or negative result for a confirmation of a case. Additionally, if such a strategy that could provide positivity solely in active pathogens’ carriers appears difficult, it would be wise to design molecular tests that discriminate between the carriers of both active and inactive pathogenic biomolecules for the better prevention of emerging infectious diseases, their illustration, and control and management at a personal and epidemiological level. 

Apart from the philosophy of the molecular diagnostic testing target, the future clinical molecular diagnostic tests ought to be multiplexed diagnostic assays that will be capable of detecting dozens of different pathogens from a sole test performance and with a more efficient processing of a clinical specimen than the current technologies to save both time and money. Outside the box of clinical molecular diagnostics, may the growing concern of bacterial resistance arising by overprescribing antibiotic drugs for non-bacterial emerging infectious diseases be solved via diagnostics such as mCARMEN. May the multiplexed assays, such as LFIAs that distinguish amongst vi-ruses, i.e., SARS-CoV-2, H1N1, etc., increase the consumers’ confidence in administering RDTs at home in the near future. Doubtlessly, these rapid multiplexed assays in future RDTs should be highly sensitive and specific for the emergency departments’ (EDs) rapid applications, so frontline physicians can provide an immediate on-the-spot diagnosis and for the management of severe cases requiring urgent acute care and treatment. 

Except for the molecular diagnostic tests that are and will be performed for the purposes of the identification of an infectious pathogenic agent, there is a tremendous variety of general molecular clinical tests that need to be evolved and novel molecular diagnostic assays to be designed, even for common blood screening tests. Previously, it was discussed that in the future, society will be highly vulnerable, there will occur higher percentages of immunosuppressed cases that will be treated with favorable effective monoclonal antibody therapies, and also there will be more well-designed vaccines that will result in the production of antibodies and, as a result, one could argue that future blood samples could be somewhat more ‘sticky’. Nevertheless, from several aspects, this fact could be critical in the future for peoples’ health. Additionally, it is already known that serologic assays are affected by the overall serum Ig levels. Serologically speaking, the most profound cause of a false-positive test is a state of hyperglobulinemia in the serum or plasma of the individual under consideration, which is the most common cause of a false-positive test; in other words, the “sticky serum”. Empirically speaking, contrary to current research on sandwich immunoassays, the falsely elevated test results can be more frequent than falsely low results because most serologic assays are taking into account the issue of a high sensitivity rather than that of a high specificity. Thus, future molecular tests for clinical diagnosis may be based on new novel assays such as combinations of immunoglobulins and nucleic acids or even some new novel (bio)molecules that will not directly face the serologic concerns. It is evident that the sandwich-like serologic methods and especially their targets need to be evolved in the near future. 

However, there arise some issues regarding the future molecular diagnostic tests. First and foremost, what factors can trammel the rapid designation, development, and deployment of molecular tests so as to identify infected cases and to what extent? Second, which policies are appropriate for the dissemination of these ARDTs in society? Third, what is the cost that is required in order to ensure the future efficient and effective testing efforts for molecular diagnostics? Finally, will it be possible for molecular diagnosis to be globally available, especially for vulnerable populaces that could be highly affected by future (re)emerging infectious pathogenic agents?

## 4. Conclusions

The COVID-19 pandemic revealed that there exist some major issues on molecular diagnostics, mainly regarding the terminology, role, quantity, and quality, during the diagnosis of emerging infectious diseases. Yet, COVID-19 taught the scientific community several lessons on molecular diagnostics. The future reality of public health will be much different from the pre-pandemic period. During the COVID-19 pandemic, society came closer together with some valuable public health maintenance policies, with the most crucial to be the application of rapid molecular diagnostics, even to a large extent, referring to self-diagnosis purposes. Yet, there exist novel plans based on preventive medicine that need to be studied and confirmed so future emerging infectious diseases, epidemics, and pandemics can be directly prevented and immediately managed. Τhe global immunological blueprint that was discussed as a novel plan for the prevention of future (re)emerging infectious diseases, epidemics, and pandemics seems to be of high scientific importance, and the investigation of common antigenic sites between pathogens can be extremely important for future vaccines, tests, and drugs as well. Communities are armored with such weapons to tackle immediately the future pandemic.
